# The Immunological Basis of Liver Allograft Rejection

**DOI:** 10.3389/fimmu.2020.02155

**Published:** 2020-09-02

**Authors:** Vincenzo Ronca, Grace Wootton, Chiara Milani, Owen Cain

**Affiliations:** ^1^Division of Gastroenterology and Centre for Autoimmune Liver Diseases, Department of Medicine and Surgery, University of Milan Bicocca, Milan, Italy; ^2^National Institute of Health Research Liver Biomedical Research Unit Birmingham, Centre for Liver Research, Institute of Immunology and Immunotherapy, University of Birmingham, Birmingham, United Kingdom; ^3^Liver Unit, University Hospitals Birmingham NHS Foundation Trust, Birmingham, United Kingdom; ^4^Department of Cellular Pathology, University Hospitals Birmingham NHS Foundation Trust, Birmingham, United Kingdom

**Keywords:** transplantation, tolerance, immunomodulatory, dendritic cells, regulatory T cell

## Abstract

Liver allograft rejection remains a significant cause of morbidity and graft failure in liver transplant recipients. Rejection is caused by the recognition of non-self donor alloantigens by recipient T-cells. Antigen recognition results in proliferation and activation of T-cells in lymphoid tissue before migration to the allograft. Activated T-cells have a variety of effector mechanisms including direct T-cell mediated damage to bile ducts, endothelium and hepatocytes and indirect effects through cytokine production and recruitment of tissue-destructive inflammatory cells. These effects explain the histological appearances of typical acute T-cell mediated rejection. In addition, donor specific antibodies, most typically against HLA antigens, may give rise to antibody-mediated rejection causing damage to the allograft primarily through endothelial injury. However, as an immune-privileged site there are several mechanisms in the liver capable of overcoming rejection and promoting tolerance to the graft, particularly in the context of recruitment of regulatory T-cells and promotors of an immunosuppressive environment. Indeed, around 20% of transplant recipients can be successfully weaned from immunosuppression. Hence, the host immunological response to the liver allograft is best regarded as a balance between rejection-promoting and tolerance-promoting factors. Understanding this balance provides insight into potential mechanisms for novel anti-rejection therapies.

## Introduction

Liver transplantation is currently the only effective treatment for end-stage liver disease. In the last 40 years the remarkable improvement in the surgical technique and the development of immunosuppressive drugs alongside improved post-transplant medico-surgical management has significantly prolonged transplant recipient survival. The host immunological response to the liver allograft is best regarded as a balance between rejection-promoting and tolerance-promoting factors. Whilst the unique features of the liver as an immunoregulatory organ promote an enhanced tolerogenic response in the allograft recipient compared with other organs, immunological rejection remains a significant clinical problem. For this reason the majority of liver transplant recipients require lifelong immunosuppression conferring an increased risk of severe complications such as infection and neoplasia (1–3). Therefore, new therapeutic strategies to induce long-term immune tolerance are required.

The majority of rejection episodes occur within the first month following transplantation and are readily amenable to treatment with high dose steroids. Acute rejection episodes can also occur in the later post-transplant period when the presentation may be less typical (4). Up to 35% of patients may experience at least one episode of acute rejection, although some will have sub-clinical disease (5). Repeated acute episodes may lead to chronic rejection. Whilst historically this was more common and occurred within a few months following transplantation, in the current era of immunosuppressive therapy the incidence of chronic rejection is probably 2–3% at most and may occur several years post-transplant (6, 7). Chronic rejection has a complex and only partly understood etiology probably representing the end stage of a number of different immunological processes (8, 9).

The objective of this review is to provide an overview of the main immunological principles governing rejection and tolerance in the liver allograft and to outline current novel therapeutic approaches aiming to induce long lasting immune tolerance after liver transplantation. Given its low incidence and complex etiology, chronic rejection will not be considered further in this review.

## Preservation-Reperfusion Injury

A certain degree of ischemic injury to the allograft is an unavoidable consequence of transplantation. This occurs during organ transportation to the transplant center (known as the cold ischemia time because the liver is transported in cold storage) and during organ harvesting and subsequent implantation (known as the warm ischemia time). An additional element of warm ischemia time is unavoidable for donation after circulatory death (DCD) as opposed to donation after brainstem death (DBD) livers because of the time lag between circulatory collapse and organ retrieval.

Ischemia leads to depletion of intracellular adenosine triphosphate particularly in hepatocytes and liver sinusoidal endothelial cells (LSEC), resulting in cell damage and death. Upon reperfusion further damage is elicited by release of reactive oxygen species and pro-inflammatory cytokines such as TNFα, IFN-γ and IL-1 by activated Kupffer cells (10). Within this acute pro-inflammatory environment LSEC are induced to upregulate cellular adhesion molecules including ICAM-1 and VCAM-1, facilitating recruitment of leukocytes to the allograft (11). Thus, the overall effect of transplantation is to induce a pro-inflammatory microenvironment within the liver allograft resulting in tissue damage, a phenomenon termed preservation-reperfusion injury (PRI, also known as ischemia-reperfusion injury) (12).

The method of organ retrieval and the presence of donor-related liver disease influence the extent of PRI related damage. The prolonged warm ischemia time of DCD livers results in exaggerated PRI principally causing additional damage to hepatocytes and resulting in inferior clinical outcomes (13). Steatotic livers are being increasingly utilized for transplantation. Steatosis is associated with increased PRI as measured by molecular markers of inflammation (14) and reflected histologically as increased hepatocyte necrosis (15). Clinically, the sequelae of PRI in DCD livers include an increased risk of primary non-function and ischemic-type biliary lesions and overall reduced graft survival (16).

## The Immunological Basis of T-Cell Mediated Rejection

**FIGURE 1:**
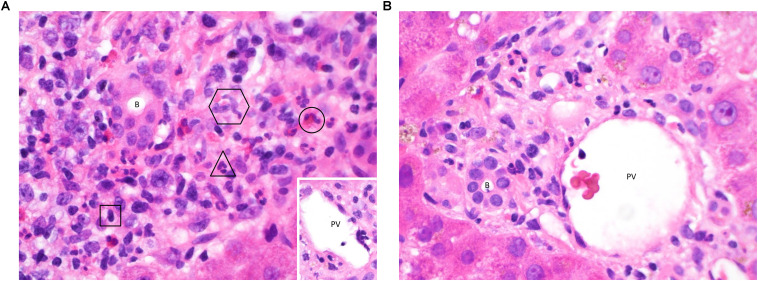
**(A)** Acute TCMR 6 days post liver transplant. This high-power image (hematoxylin & eosin (H&E), ×600) of a portal tract shows infiltration of a large number of inflammatory cells. Typical examples of a lymphocyte (square), neutrophil (triangle), eosinophil (circle) and two macrophages (hexagon) are highlighted, demonstrating the contribution of cells from both the innate and adaptive immune systems. The majority of the lymphocytes will be T-cells, capable of mediating cell damage through direct cytotoxicity and the release of pro-inflammatory cytokines. There is clustering around the bile duct (B) and portal vein (PV, inset, with damage to endothelium seen at 4 o’clock). Neutrophils and macrophages migrate to the liver in response to pro-inflammatory cytokines, enhanced by Th1 and Th17 responses. Eosinophils are also present in early rejection infiltrates and are more typically associated with a Th2 response. Treatment with high dose pulsed methylprednisolone was able to suppress the rejection episode in this patient and tolerance has since been maintained using a standard immunosuppressive regimen. **(B)** Immune tolerance 8 days post liver transplant. In contrast to Figure 2A, a portal tract from this biopsy (H&E, ×600) contains only a small number of lymphocytes, macrophages and neutrophils with no evidence of damage to biliary epithelium (B) or portal vein endothelium (PV). Tolerance to the liver allograft is promoted by multiple factors including the relatively low levels of MHC class II expression on hepatic-resident cells, a tendency toward tolerogenic antigen presentation by the dendritic cells, macrophages, stellate cells and epithelial cells resident in the liver, the dominance of a regulatory T-cell infiltrate and the action of donor-derived NK cells on the recipient immune system. Indeed, a biliary complication of surgery was found to be the reason for liver dysfunction in this patient, and tolerance was maintained on follow-up by means of a standard immunosuppressive regimen without the need for additional therapy.

T-cell mediated rejection (TCMR, also previously known as “acute cellular rejection”) occurs most commonly in the early post-transplant period and is generally amenable to treatment with immunosuppression (17). It typically presents with non-specific clinical symptoms and predominantly cholestatic liver biochemistry. Liver biopsy is required for diagnostic confirmation and shows a dense portal-based mixed inflammatory cell infiltrate with evidence of damage to biliary epithelium, portal and hepatic vein endothelium and hepatocytes (18) (Figure 1). Early episodes of TCMR do not impact on long-term outcomes (19) although persistent rejection episodes refractory to standard therapies remain problematic. This section of the review will outline our current understanding of the immunological mechanisms that give rise to TCMR and the mechanisms of allograft damage elicited by the cellular infiltrate.

### Major Histocompatibility Complex Antigen Expression

The main antigens responsible for driving rejection are the major histocompatibility complex (MHC) molecules. MHC class I molecules are constitutively expressed by all nucleated cells and present intracellular epitopes to CD8 + cytotoxic T-cells. In contrast, expression of MHC class II molecules is more restricted, presenting epitopes derived from extracellular material to CD4 + helper T-cells. In the normal liver there is strong and diffuse MHC class I expression in all cells whereas MHC class II expression is limited to Kupffer cells and other liver-resident antigen presenting cells. During liver inflammation expression of MHC class I is increased in all cells and MHC class II expression is stimulated in endothelium, biliary epithelium and hepatocytes (20). Thus, liver inflammation upregulates expression of MHC molecules, priming toward a rejection response.

### Preservation-Reperfusion Injury

PRI has long been recognized as important factor in skewing the recipient immunological response in favor of rejection (21). Damaged hepatocytes and LSEC release damage-associated molecular pattern molecules (DAMPs): HMGB1, free fatty acids and heat shock proteins. This activates Kupffer cells via toll-like receptors, stimulating release of pro-inflammatory cytokines such as IL1, TNF, IFN and IL12. Release of CXCL-1, -2 and -3 stimulates neutrophil recruitment to the graft (22). PRI also promotes upregulation of lymphocyte recruitment molecules by LSEC. The end result of PRI is therefore the establishment of a pro-inflammatory microenvironment within the liver.

PRI may promote TCMR by providing the initial stimulus for migration of donor-derived dendritic cells (DC) from the transplanted liver to recipient regional lymph nodes. These professional antigen presenting cells are resident in the liver and upregulate expression of MHC class I and II molecules as a consequence of the inflammatory signals generated by PRI. The chemotactic PRI signals also act as a means of recruiting activated T-cells of the adaptive immune system and to amplify their rejection-mediating effects.

### Alloantigen Presentation, T-Cell Activation and Maturation

**FIGURE 2:**
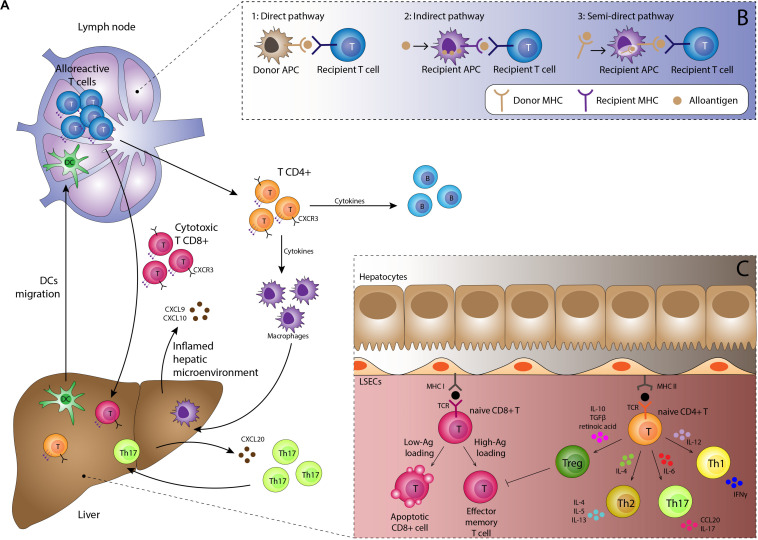
**(A)** Immunological basis of TCMR. Activated dendritic cells migrate to lymphoid tissue presenting alloantigen on MHC class I and II molecules. Interaction with naïve alloreactive T-cells in the presence of appropriate co-stimulatory molecules and a pro-inflammatory cytokine milieu results in proliferation of alloreactive CD4 + and CD8 + effector T-cells and subsequent B-cell proliferation. Migration to the liver is orchestrated by chemokines such as CXCL9 and CXCL10 interacting with the CXCR3 receptor on lymphocytes in addition to complex interactions with the unique immunomodulatory liver sinusoidal endothelial cells. Lymphocyte subsets such as Th17 cells have specific recruitment mechanisms, providing potential therapeutic targets. Cells of the innate immune system including macrophages, neutrophils and eosinophils are recruited to the liver and along with the effector T-cells mediate tissue damage, resulting in the clinical manifestations of TCMR. **(B)** Three pathways for antigen presentation. The strongest alloimmune response is generated by the direct pathway which occurs in the early post-transplant period. Upon activation, donor-derived dendritic cells migrate to the lymph node, displaying non-self donor antigens within non-self donor MHC molecules. This provides potent stimulation for the mounting of a rejection response. The indirect and semi-direct pathways involve recipient-derived dendritic cells displaying self and non-self MHC molecules respectively. Whilst not as potent as the direct pathway, they are still able to sustain ongoing rejection. **(C)** Antigen presentation within the liver generally promotes tolerogenic responses. Antigen is also presented within the allograft by endothelial cells, macrophages, hepatic stellate cells, hepatocytes and biliary epithelium, with increased presentation seen during episodes of inflammation. However, most interactions with naïve lymphocytes within the liver result in tolerance rather than rejection, with apoptosis of effector cells and a skewing of T-cell differentiation toward the regulatory T-cell phenotype through an immunosuppressive cytokine profile.

Alloantigen presentation by DC is a key step in rejection. In the normal liver DC are present in portal tracts and around hepatic veins, and thus significant numbers of donor-derived DC are transferred to the recipient as passengers during transplantation. In response to pro-inflammatory environments such as PRI they become activated, upregulate expression of MHC molecules displaying alloantigens and mobilize to lymphoid tissue (20). Activated donor-derived DC arriving in the lymph node provide a potent immunological stimulus for recipient-derived naïve CD4 + T-cells, which recognize as foreign not only the presented antigen but also the MHC molecule itself, known as the direct pathway of antigen presentation (Figure 2). The interaction between the DC and T-cell is dependent on: (1) activation of the T-cell receptor (TCR) by its cognate peptide-MHC complex on the DC, (2) interaction between T-cell integrin adhesion molecules such as LFA-1 and VLA4 interacting with ICAM-1 and VCAM-1 on DC, and (3) co-stimulatory molecule interactions such as CD28 expressed on T-cells interacting with B7 molecules on DC. Since dendritic cells express MHC class I and class II molecules they are able to activate both CD4 + and CD8 + T-cells (23). Successful priming of naïve T-cells leads to activation of the cytoplasmic calcium-dependent phosphatase enzyme calcineurin within T-cells, which in turn activates nuclear transcription factor of activated T-cells (NFAT), upregulating expression of IL-2. This cytokine provides the main stimulus for T-cell proliferation by interacting with the cell surface IL-2 receptor.

The indirect and semi-indirect pathways are more typically associated with later episodes of rejection. These pathways are mediated by recipient (as opposed to donor) DC, which accumulate within the graft over time. The indirect pathway is characterized by alloantigens captured and processed by recipient DC or other antigen presenting cells and then presented upon self-MHC molecules to naïve T-cells (24). The semi-indirect presentation refers to the expression of the intact donor MHC on the surface of the recipient antigen presenting cells. The semi-indirect pathway is considered to be of particular importance in allograft rejection and is probably the consequence of a cell to cell contact and the fusion of recipient and donor exosomes (25–29). Whilst still capable of initiating a rejection response, the indirect and semi-indirect pathways are less potent than the direct pathway.

Once primed, CD8 + T-cells predominantly differentiate into cytotoxic T-cells (Tc) able to exert direct cell damage on the allograft. CD4 + T-cells have the potential to differentiate into a number of activated subtypes, of which the helper T-cell (Th1, Th2 and Th17) and regulatory T-cell (Treg) subsets are the best characterized. The relative proportion of cells in each subtype is determined by the local inflammatory microenvironment. In acute rejection T-cell differentiation is primarily polarized toward the Th1 response, driven by pro-inflammatory cytokines such as IL-12, TNF-β and particularly IFN-γ. Th1 cells are characterized by secretion of IL-2 and IFN-γ which provide a positive feedback loop stimulating further proliferation of Th1 cells.

Whilst the Th2 response was initially characterized as immunosuppressive, it is now recognized to mediate acute rejection, at least under certain circumstances (30). Differentiation toward the Th2 phenotype is promoted by IL-4; Th2 cells themselves then produce IL-4 and IL-5 providing another example of a positive feedback loop (31). Th17 cells also play a role in acute TCMR. However, the relationship between Th1 and Th17 differentiation remains unclear as Th17 differentiation is inhibited by IFN-γ (32). Th17 differentiation is however, provoked by pro-inflammatory mediators such as Il-1, IL-6, IL-21 and IL-23 and TGF-β, prostaglandin E2 and HMGB-1 (33).

Activated lymphocytes must migrate toward and gain access to the liver in order to carry out their effector functions. A pro-inflammatory microenvironment in the allograft promotes endothelial secretion of IFN-γ inducible chemokines, namely CXCL9 and CXCL10, which facilitate the attraction of circulating leukocytes, including activated T-cells, expressing the chemokine receptor CXCR3 (34). Leukocyte migration across target organ endothelium typically follows a sequential process of (1) tethering, (2) activation mediated by LFA-1/ICAM-1 and VLA-4/VCAM-1 interactions and (3) crawling/transmigration through the endothelium to gain access to the liver (35). However, the main site of leukocyte recruitment in the liver is within the sinusoids, mediated by LSEC, which possess a number of unique immunomodulatory functions resulting in non-classical mechanisms of lymphocyte recruitment (36). For example, whereas CD8 + T-cell recruitment is largely mediated by ICAM-1 (37, 38), Treg recruitment also involves molecules such as stabilin-1 and VAP-1 (39). Neutrophil recruitment across LSEC is independent of the selectin-mediated interactions known to be important at other sites, instead relying on interactions between LSEC-produced hyaluronan and neutrophilic CD44 (40). Furthermore, VAP-1 has been shown to mediate lymphocyte migration across LSEC in an animal model of TCMR (41). Manipulation of the immunological properties of LSEC therefore provides a potential opportunity to shape the immune response to the allograft.

### Effector Responses

#### CD8+ Tc Cells

Primed CD8 + Tc cells are the main effector lymphocytes responsible for mediating tissue damage. This process depends on the binding of the TCR to the non-self donor-derived MHC class I molecules widely expressed on biliary epithelial cells (BEC), endothelium (portal, sinusoidal and centrilobular) and hepatocytes. Activation of cytolytic activity is dependent on interactions of cell adhesion molecules such as LFA1-ICAM1 and CD2-LFA3 as well as the TCR-MHC-peptide complex. The cytolytic activity of Tc cells is mediated through two main pathways: (1) the granzyme/perforin pathway in which the pore-like perforin molecule is released from the T-cell, punctures the cell membrane of the target cells facilitating entry of granzymes to the target cell cytoplasm which initiates apoptosis and (2) the Fas-FasL pathway in which activation of the Fas molecule on the surface of target cells by its ligand FasL on Tc cells leads to activation of the death domain in the cytoplasmic tail of Fas and caspase-dependent apoptosis.

Hepatocytes are relatively resistant to Fas-FasL mediated damage. Instead, other molecules of the TNF superfamily receptors such as CD40, TRAIL and TNFR1-2 which fulfill a similar role appear to be more important. Their expression is upregulated on the surface of BEC and hepatocytes during inflammation, facilitating Tc-mediated cell death (42–44). Interestingly, there is emerging evidence that initial Tc-target cell interactions may occur via cytoplasmic protrusions extending from intra-sinusoidal T-cells (45).

#### CD4 + Th Cells

The pro-inflammatory Th1 response is considered to be the main driver of acute TCMR. Continued production of IL-2 and IFN-γ by Th1 cells is important for macrophage activation and ongoing stimulation of CD8 + Tc cell subsets, which produces further IFN-γ, acting as a positive feedback loop (33). Th1 subsets also cause allograft damage directly through Fas-FasL mediated cytotoxicity in the same manner as Tc cells (46).

Th1 and Th2 responses have an antagonistic relationship such that production of the Th2 cytokines IL-4 and IL-10 inhibits Th1 differentiation. Indeed, there is some evidence that under certain circumstances a Th2 polarized response is tolerogenic in the liver allograft (47). However, there is also considerable evidence implicating Th2 cells as direct mediators of rejection (30, 48, 49). Mechanisms include interaction between Th2 cells and activated B-cells leading to the production of donor specific antibodies, with proliferation of activated B-cells stimulated by IL-2 production by Th1 cells, illustrating the cross-over between cell- and antibody-mediated rejection. Th2 responses are also important for the recruitment of eosinophils, which are present in abundance in early TCMR.

Th17 cells exert tissue damaging functions by virtue of IL-17 production which acts as a powerful signal for neutrophil recruitment. Th17 cells are able to promote liver allograft rejection in a rat model (50) and high levels of peripheral blood Th17 levels have been associated with impaired tolerance in clinical studies (51). The CXCR3 receptor has been shown to be critical for Th17 cell migration into the inflamed liver; the cells then home to portal tracts with particular tropism toward BEC expressing the CCR6 ligand CCL20 (52). Subsequent work has shown the active role of BEC in maintaining Th17 dominant differentiation via release of IL-6 and IL-1β, and the stimulation of BEC proliferation by Th17 cytokines (53). There appears to be a degree of plasticity between Th17 and Treg differentiation such that the two exist in a state of dynamic equilibrium; this has generated interest in the importance of these divergent populations in skewing the immune response toward rejection or tolerance (see below).

#### Memory T-Cells

Following initial presentation of a novel antigen, a small number of T-cells differentiate into long-lived memory T-cells rather than effector cells. Memory cells reside in peripheral tissues and are able to respond more rapidly and potently than naïve T-cells on repeat exposure to the antigen. One of the main mechanisms for this enhanced response is the reduced requirement for CD28-B7 co-stimulatory signals.

Counterintuitively, memory T-cells have been shown to play a key role in the initial acute allograft rejection response as well as in later episodes of TCMR despite the fact that the allograft is “new” to the recipient (54). Potentially alloreactive memory T-cells can be demonstrated in the serum of healthy volunteers (55) and higher numbers of alloreactive pre-transplant memory T-cells correlate with an increased risk of post-transplant rejection episodes (56). Potential mechanisms for the generation of immunological memory in the pre-transplant population include:

• Historical direct exposure to alloantigen via pregnancy or blood transfusion.
• Heterologous immunity in which there is cross-reactivity between a previously encountered pathogen-related antigen and allogenic peptides.
• Homeostatic proliferation following lymphodepletion by pharmacological immunosuppression. During this process surviving T-cell to undergo homeostatic proliferation and differentiation into “pseudo memory T-cells” despite never having been presented with antigen (57).

Memory T-cells of the CD4 + helper class have the potential to induce antibody mediated rejection via enhanced production of donor specific antibodies by B-cells whereas CD8 + memory T-cells are able to exert direct cytotoxic effects. Memory T-cells are less sensitive to immunosuppressive treatments compared with naïve T-cells and could be one reason why some patients do not fully respond to standard treatments for acute TCMR. As such memory T-cells are a potential barrier to establishing tolerance and their impact on rejection requires further study (58).

#### B-Cells

B-cells are not generally discussed in the context of TCMR. However, B-cell deficiency in mice and humans has been associated with delayed acute rejection (59). Potential mechanisms include the activation of T-cells by B-cells via costimulatory pathways and cytokine release and promoting T-cell differentiation into memory T-cells (60). B-cell presentation of donor antigen is enhanced during liver allograft rejection and may provide a novel target for immunosuppression (61). The main role of B-cells is however, the production of antibody which is of key importance for antibody mediated rejection.

#### Macrophages

The macrophage response is often conceptualized as being either pro-inflammatory, stimulated by IFN-γ and lipopolysaccharide (the so-called M1 phenotype) or immunosuppressive, stimulated by IL-4 and IL-13 (the M2 phenotype). In acute rejection many macrophages show features of polarization toward an M1 phenotype producing pro-inflammatory cytokines such as IL-1, IL-12, IL-18, IL-6, IL-23, TNF-α and IFN-γ and reactive oxygen and nitrogen species which cause direct cell damage and co-ordinate a pro-inflammatory immune response (62). Recognition of damaged allograft tissue is through the pattern recognition receptors such as the toll-like receptors and macrophages have a major phagocytic role in the clearing of damaged cells (63). As antigen presenting cells intrahepatic macrophages are able to present alloantigens in MHC class II molecules, thus promoting the adaptive immune response. Unsurprisingly an M1 macrophage response has been associated with allograft rejection (64) whereas an immunosuppressive M2 response is associated with tolerance (65). Early M1 macrophages have been shown to differentiate into M2 macrophages following loss of co-stimulatory signals (66). Macrophage polarization is mediated by a number of cytokines and growth factors (67).

The M1/M2 framework for understanding macrophage responses is however, an over-simplification. Whilst different macrophage populations certainly possess divergent functions, understanding macrophage biology is complicated by the replacement of donor derived macrophages in the early post-transplant by recipient derived cells differentiating from circulating monocytes in the later period (68). Furthermore, the phenotypic diversity of macrophage subsets within the liver does not readily permit a binary classification (20). However, attempts at further delineating the pathways involved in producing a more immunosuppressive macrophage response are likely to feed into therapeutic efforts to identify novel anti-rejection therapies.

#### Neutrophils

Neutrophils are often numerous in acute TCMR and may be recruited to the allograft following PRI and as an early effector response to adaptive alloimmunity, particularly in response to Th17 activation. Neutrophils mediate cell damage via ROS generation, numerous tissue-digesting enzymes such as metalloproteinase-9 and neutrophil elastase (69), and possibly through a unique form of programmed cell death (70). As classical mediators of the acute inflammatory response, neutrophils may also play a role in tipping the immunological balance toward rejection following an episode of infection (71). Intriguingly, neutrophils may also have a role to play in tolerance mechanisms, having been shown to have the capability to inhibit T-cell responses (72) and polarize macrophages toward a M2 phenotype in an animal model (73).

#### Eosinophils

In contrast to macrophages and neutrophils, which respond primarily to a classical pro-inflammatory Th1 response, eosinophil maturation and migration is orchestrated by Th2 cytokines such as IL-4 and IL-5. Eosinophils have long been recognized as a key feature of TCMR in the liver (74) and peripheral eosinophilia has been associated with rejection (75). Cell damage is mediated by secretion of cytotoxic granules including major basic protein which increases permeability of cell membranes and eosinophil peroxidase. Of interest, eosinophils also have receptors for Th1-associated cytokines such as TNFα (76) and recruitment may therefore not be entirely dependent on Th2 pathways.

#### NK Cells

Natural kill (NK) cells are lymphocytes that lack expression of CD3, CD20 and other typical T- and B-cell markers, instead expressing CD16 and CD56. NK cells can be stimulated by both activating signals and the loss of inhibitory signals. In the allograft potential activating signals come from molecules such as MIC-A and MIC-B expressed by allograft tissue as a stress response to a pro-inflammatory environment (77). These molecules are recognized by activating receptors on NK cells such as NKG2D. Inhibitory signals come from self MHC class I molecules which normally interact with the inhibitory receptors on NK cells such as killer immunoglobulin-like receptors (KIRs). In rejection it is postulated that the non-self MHC class I molecules present on the cells of the allograft are unable to maintain the inhibitory KIR signal (78). As such solid organ allografts provide multiple mechanisms for activation of recipient NK cells that have migrated to the graft.

NK cells are able to mediate cytotoxicity through production of perforin and granzyme in a similar manner to Tc cells. Activated NK cells and also produce INF-γ and TNF-α promoting early adaptive immune responses and further tissue damage, an effect demonstrated to be of importance in a rat model of liver transplantation (79). NK cells also have the ability to recognize antibody on target cells using Fc receptors, linking the NK response to antibody mediated mechanisms.

However, many of the pathways linking recipient NK cells with rejection remain unclear. A clinical study matching KIR and MHC class I types did not impact upon allograft rejection or clinical outcome (80). Whilst they are most likely of importance, the precise mechanisms of NK-cell mediated rejection requires further clarification.

Further populations of unconventional T-cells such as NK T-cells and gamma delta T-cells may also play a role in rejection and tolerance mechanisms, although at present an understanding of their importance in the allograft is limited (81, 82).

## The Immunological Basis of Antibody Mediated Rejection

The most severe form of antibody mediated rejection (AMR) is hyperacute rejection which occurs in ABO-incompatible grafts and is vanishingly rare in the liver. It results in acute liver failure within hours to days (83). In contrast to other solid organ transplants, the clinical significance of other forms of AMR in the liver was initially unclear, but it is now generally accepted that antibodies can mediate clinically significant rejection episodes (84). Isolated acute AMR in the liver is rare, has a clinical presentation that overlaps with TCMR and may often quickly evolve into TCMR (85). Furthermore, biopsy findings are not specific and a diagnosis of AMR requires correlation with clinical, serological and immunohistochemical data. The immunological basis of AMR is however, reasonably well characterized, largely based on data from other solid organ transplants, particularly the kidney.

### Antibody Production

Donor specific antibodies (DSA) capable of causing AMR may be either pre-formed or arise *de novo* post-transplant. The presence of preformed alloantibodies can be explained by similar mechanisms as those for pre-existing memory T-cells discussed above. *De novo* antibody production occurs when naïve B-cells interact with alloantigens (mainly MHC molecules) via the B-cell receptor following classical adaptive immunological pathways. In the presence of inflammatory signals such as IL-1 this leads to B-cell activation, internalization and degradation of the antigen by the B-cell and re-presentation of antigen fragments by MHC class II molecules. These molecules are able to directly interact with primed Th2 cells in an indirect manner of antigen presentation (86). When co-stimulatory and cell adhesion signals such as CD28-B7, CD40L-CD40, LFA-1-ICAM and CD2-LFA-3 are also activated then B-cell division and differentiation can occur. This process is facilitated by IL-2 production from Th1 cells, in addition to Th2 cytokines such as IL-4 and IL-5. Some activated B-cells differentiate into plasma cells and begin production of DSA. Other cells migrate to lymph nodes forming germinal centers and undergo a process of somatic hypermutation and affinity maturation, refining and amplifying the antibody response. Mature plasma cells are able to produce antibodies indefinitely without T-cell help (87). Memory B-cells are also produced facilitating ongoing episodes of rejection.

### Antibody Effector Functions

**FIGURE 3:**
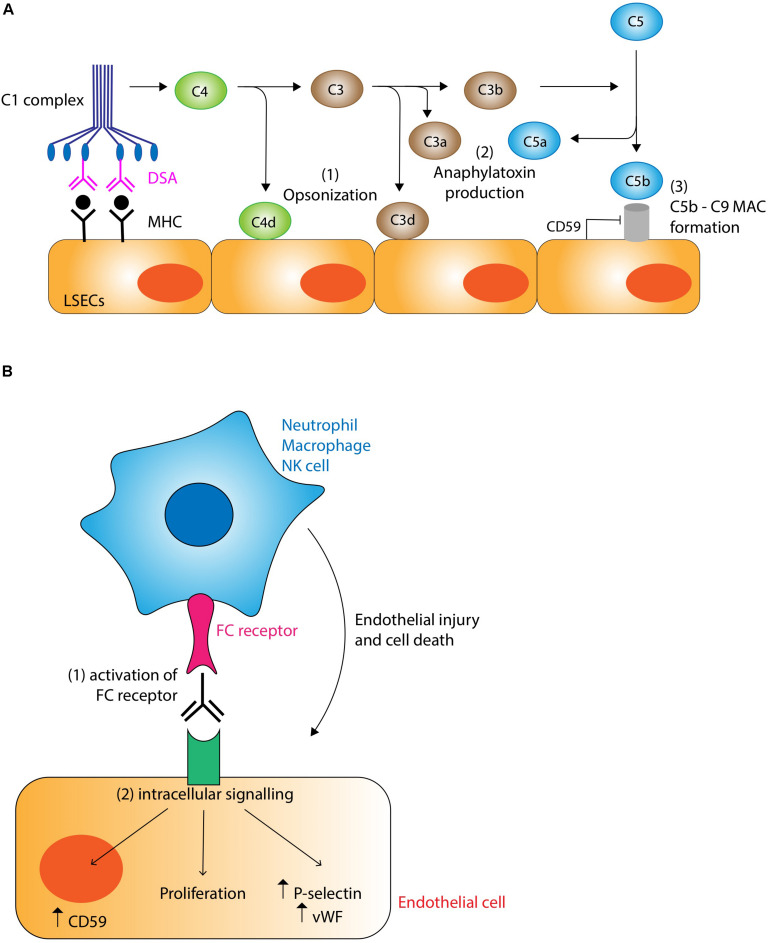
**(A)** Complement-dependent mechanisms of antibody mediated rejection. Binding of donor specific antibody (DSA) to MHC molecules on the liver allograft causes activation of the classical pathway of complement via binding of the C1 complex. Complement has the potential to damage the graft through three main mechanisms: (1) Opsonization. C4d and C3d covalently bind to target cells marking them for destruction and clearing by cells of the innate immune system. (2) Anaphylatoxin production. C3a and C5a act as potent chemotactic signals recruiting inflammatory cells which cause localized tissue damage. (3) Membrane attack complex (MAC). The C5b-9 MAC has the potential to damage cells by puncturing holes in the membrane, although this action is normally inhibited by endothelial expression of CD59 (protectin). Non-lytic binding of the MAC induces endothelial upregulation of pro-inflammatory, lymphocyte recruitment, and MHC molecules, thus potentiating the rejection response. **(B)** Complement-independent mechanisms of antibody mediated rejection. DSA binding to MHC molecules promotes the recruitment of cells of the innate immune system such as neutrophils, macrophages and NK cells via interactions with the FC receptor. These inflammatory cells are stimulated to cause graft injury via their various effector mechanisms (see text for details). DSA binding also stimulates intracellular signaling pathways.

The main targets of DSA are the non-self class I and II MHC molecules expressed by endothelial cells within the liver allograft, the latter being significantly upregulated by pro-inflammatory signals. Anti-MHC class I antibodies tend to appear earlier, while anti-MHC class II antibodies (particularly anti-HLA-DQ antibodies) develop in the later post-transplant period (88). Interaction between DSA and their target antigen causes activation of the classical pathway of the complement system via the binding of C1q to the Fc regions of bound DSA (Figure 3A). This initiates an enzyme cascade producing biologically active complement effector functions. Although the role of these mediators in AMR has not been fully elucidated in the liver, chemotactic signals such as C3a and C5a are potent inflammatory mediators (anaphylatoxins) likely to be important for activating mast cells and basophils and recruiting macrophages and granulocytes including eosinophils, macrophage activation and increasing vascular permeability (89). Production of C3d opsonizes target cells by covalent bonding promoting phagocytosis. C5b forms the membrane attack complex C5b-9 with the potential to cause direct endothelial damage via puncture of the cell membrane with the pore, although expression of CD59 (also known as protectin) may provide endothelial cells with some resistance to this form of injury (90). The non-lytic binding of the C5b-9 complex to the endothelial surface also induces the expression of several pro-inflammatory proteins including IL-6, E-Selectin, and VCAM-1, and upregulates expression of IFN-γ and MHC molecules endothelial cells further amplifying the antibody response (91). Complement also interacts with the adaptive immune system, augmenting T-cell mediated rejection (92). Immunohistochemical demonstration of C4d deposition on allograft vasculature is used as a marker of complement system activation and AMR. C4d is a product of C4b degradation and is a more sensitive marker of antibody binding than direct measurement of immunoglobulin deposition because C4d shows covalent bonding to the endothelial surface and amplifies the immunoglobulin signal.

Although complement appears to be the main mechanism of tissue damage in AMR, it is increasingly recognized that complement-independent pathways are also important (Figure 3B). One mechanism involves the binding of Fc receptors on neutrophils, macrophages and NK cells to bound DSAs. The resulting activation of these cells of the innate immune system triggers a cascade of pro-inflammatory pathways leading to endothelial cell damage (90). Another complement-independent mechanism involves the direct activation of intracellular signaling pathways within endothelial cells by the binding of DSA to MHC molecules, resulting in structural changes to cytoskeletal proteins, increased cellular proliferation, increased production of von Willebrand factor and P-selectin and the enhanced expression of CD59 conferring resistance to C5b-9 mediated attack (88, 93).

Thus the end result of DSA binding to allograft endothelium is endothelial damage and swelling, formation of microthrombi, platelet aggregates and inflammation. In acute AMR of the liver these changes generally manifest as portal edema and hemorrhage, bile ductular reaction, and dilatation of portal microvessels (94). Portal eosinophilia, eosinophilic central venulitis and portal microvessel endothelial hypertrophy and “hobnailing” have been identified as more specific features (95).

## Tolerance Mechanisms in the Liver Allograft

**FIGURE 4:**
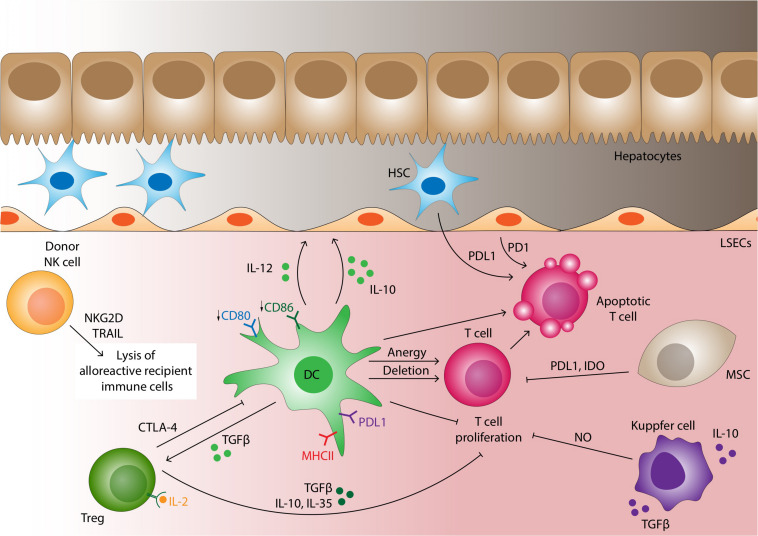
Cellular mechanisms of liver allograft tolerance. A number of cells are able to promote tolerance in the liver allograft. Under normal non-inflamed conditions dendritic cells express low level of co-stimulatory molecules which along with the high expression of PDL1 induces T-cell anergy or deletion of the alloreactive T-cell clone. DCs also promote tolerance by secreting IL-10 and TGF-β which induces the differentiation of Tregs. CTLA4 on Tregs surface binds B7 on DC with a higher affinity than CD28, impairing DC-T-cell interactions. Tregs also contribute to the tolerogenic microenvironment also secreting TGF-β, IL-10 and IL-35, binding IL-2 on CD25 with higher affinity then T effector cells and by direct cytotoxicity through granzyme, perforin and Fas-FasL pathway. In contrast to recipient derived NK cells which tend to mediate rejection, donor derived NK cells transplanted as passenger cells within the liver allograft are able to directly lyse alloreactive recipient immune cells via NKG2D-MIC-A and TRAIL-TRAILR interactions leading to caspase-induced cell death. Mesenchymal stromal cells (MSCs) suppress T-cell proliferation and differentiation through the IDO pathway and cell-cell contact mediated by PDL1. Kupffer cells may be polarized to the M2 phenotype producing IL-10 and TGF-β and thus promoting tolerance. They can also release NO if stimulated by IFN-γ to inhibit T-cell proliferation. LSEC acts as non-professional antigen presenting cells with generally low levels of MHC class II expression; under many conditions induces antigen-specific tolerance. LSEC along with hepatic stellate cells induce T-cell apoptosis through PDL1/PD1 pathway interactions.

Allograft tolerance is mediated by immunological dampening and inhibition of the rejection response (Figure 4). The liver is considered relatively tolerogenic compared with other solid organ transplants, allowing routine transplantation of non-HLA matched organs. “True tolerance” occurs when there is no demonstrable immunological response to the allograft and is a rare event (96). Nonetheless, 20% of adults and up to 65% of pediatric liver allograft recipients can exhibit preserved graft function for at least 1 year after weaning immunosuppression, despite many showing persistent subclinical immunological markers of rejection (97, 98); this is referred to as “operational tolerance.”

The explanation for the relative tolerogenicity of the liver is multifactorial: (1) The large size of the organ results in a far greater endothelial surface area over which antibodies are diluted, thus attenuating their effects, (2) The liver has an inherent regenerative capacity such that tissue destruction by episodes of rejection is potentially reversible, (3) Expression of MHC class II molecules on liver cells is variable compared with the constitutive expression seen in kidneys and hearts, (4) cell-specific mechanisms operate to attenuate the rejection response, as discussed below. Enhanced tolerance in the liver has an evolutionary basis since 75% of hepatic blood flow is from the portal vein which collects blood from the gastrointestinal tract enriched with microbial antigens. Thus, the hepatic immune system has evolved to tightly regulate immune reactions to harmless gut-derived micro-organisms in order to avoid inappropriate pro-inflammatory responses. These mechanisms are of importance for allograft tolerance.

### Mesenchymal Stromal Cells

Mesenchymal stromal cells (MSCs) are localized in the liver and although they are yet to be fully characterized, their ability to modulate the immune response is well recognized (99). These cells can be found in the perivascular space of virtually all organs (100). It is interesting to compare two highly tolerogenic organs, both extremely vascularized, such liver and placenta. Intriguingly in both the organs MSCs seem to play a decisive role in maintaining tolerance. In a similar manner to the tolerance required in the liver for gut-derived micro-organisms, the placenta needs to maintain a tolerogenic environment to allow the fetus to develop. During pregnancy several immunoregulatory mechanisms ensure the protections of the fetus which expresses paternal antigens, recognized as non-self by maternal immune system. MSCs can interact with APCs to re-program them toward a tolerant phenotype as evident from increased IL-10 secretion. Moreover, they can modulate the co-stimulatory signal on DCs inducing pro-stimulatory functions (99). MSCs can reduce the activity of T-cells using different mechanisms, for example by secretion of indoleamine 2,3-dioxygenase (IDO), an enzyme capable of metabolizing the amino acid tryptophan to kynurenine. T-cells rely on this amino acid to become activated, and the depletion of tryptophan induces apoptosis or inhibiting their proliferation and differentiation by cell to cell contact, a process mediated by PD-L1 (101, 102).

### Tolerogenic Antigen Presentation

Successful T-cell activation and differentiation depends upon the presence of co-stimulatory molecules such as CD28-B7 and a pro-inflammatory microenvironment mediated by cytokines such as IL-12. In the absence of these factors alloantigen presentation leads to anergy, a state in which the T-cell is unable to mount an effector response, and cell death.

Whilst DC antigen presentation is a major driver of rejection, a range of tolerogenic DC phenotypes have also been identified in the liver (103, 104). These cells are characterized by low levels of MHC class II and co-stimulatory molecule expression, low levels of IL-12 production, and high levels of IL-10 production; the latter stimulating Treg differentiation and inhibiting production of pro-inflammatory cytokines by macrophages (105, 106). Furthermore, some DC subtypes express PD-L1, which binds to PD-1 on T-cells inhibiting the CD28-B7 co-stimulatory signal and arresting T-cell maturation (107–109). Macrophage colony-stimulating factor (110) and hepatocyte growth factor (111) favor differentiation toward tolerogenic DC whereas FLT3L is associated with an activated alloreactive phenotype (112). Tolerogenic DC have been demonstrated in secondary lymphoid tissue following liver allograft (113) and upregulation of MHC class II and B7 in these cells leads to rejection (114). The possibility of harnessing this mechanism with pre-transplantation infusion of donor-derived tolerogenic DC is now being explored in the clinical setting (115).

Kupffer cells, LSEC, hepatocytes and hepatic stellate cells also have the ability to express MHC class I and II, particularly in pro-inflammatory states, and thus have the potential to activate alloreactive T-cells (103, 116, 117). However, in common with tolerogenic DC, non-professional antigen presentation in the liver tends to lack sufficient co-stimulatory B7 expression, to over-express PD-L1 and PD-L2 and to produce IL-10 and TGF-β, thus favoring tolerogenesis (118, 119). In support of these observations, T-cell activation in lymphoid tissue is generally much more potent than in the liver (120). However, under conditions of high antigen load local hepatic antigen presentation is able to overcome tolerogenic barriers and successfully stimulate CD8 + Tc activation (121).

### Regulatory T-Cells

Regulatory T-cells (Treg) are a population of T-cells that suppress immune responses and maintain immune homeostasis and self-tolerance. They differentiate either in the thymus or in the periphery and multiple subtypes have been described, of which the CD4 + /CD25 + /FOXP3 + is the prototypical example (122). There is a close reciprocal relationship between Treg and Th17 differentiation: TGF-β in the absence of pro-inflammatory cytokines induces FOXP3 expression and Treg differentiation, whereas if pro-inflammatory cytokines are also present then TGF-β induces Th17 differentiation (123). Treg control effector T-cells via several distinct mechanisms: (1) production of immunosuppressive cytokines such as IL-10, TFG-β and IL-35, (2) consumption of IL-2 via the Treg CD25 receptor, thus depriving activated T-cells of the main driver of proliferation, (3) direct cytotoxicity via granzyme/perforin and Fas-FasL-dependent pathways and (4) constitutive Treg expression of CTLA-4 which acts as an alternative inhibitory ligand for B7 on DC with a higher affinity than the co-stimulatory molecule CD28, thus impairing DC-T-cell interactions (124).

The importance of Treg in liver transplantation has been demonstrated through a liver allograft model in which tolerant mice treated with Treg depleting anti-CD25 antibodies experienced rejection with a reduced Treg/T-effector cell ratio (125). Moreover, animal models have demonstrated Treg stimulated *in vitro* with alloantigens capable of inducing long-term tolerance (126). In clinical studies increased numbers of circulating Treg are associated with tolerance of the allograft liver (127). Treg are also enriched in operationally tolerant liver allograft recipients (128).

### Activation-Induced Deletion of Recipient Effector Lymphocytes

The liver retains tolerogenic potential even when tissue destructive alloreactive T-cells have gained access to the parenchyma and begun to mediate tissue damage. Several groups have demonstrated that such alloreactive T-cells undergo cell death either via apoptosis (129, 130) or lysosome-mediated degradation by hepatocytes (131). This process is at least partially dependent on Treg (125) and provides a mechanism for modulating the rejection response into one of tolerance.

### NK Cells

NK cells are a major component of the resident lymphoid cell population in the normal liver (132). As such, the transplanted liver contains a significant population of donor-derived NK cells, which have been shown to persist for up to 2 years post-transplant (133). In contrast to recipient-derived cells, donor-derived NK cells do not lose the inhibitory KIR-MHC class I signal upon interaction with donor cells, instead being potentially activated by infiltrating recipient-derived leukocytes. In line with this hypothesis, expanded NK cell populations have been identified in liver transplant patients successfully weaned from immunosuppression (134). Donor-derived NK cells cause direct lysis of alloreactive recipient immune cells via NKG2D-MIC-A and TRAIL-TRAILR interactions leading to caspase-induced cell death (135). Hence, recipient-derived NK cells are likely to mediate rejection whereas donor-derived NK cells are likely to be tolerogenic (135). However, the situation may be complicated by the emergence of tolerogenic recipient-derived NK cell populations, arising through mechanisms such as dysregulation of the IL-12/STAT4 pathway (136).

### Chimerism

Chimerism is defined as the presence of donor-derived cells within non-transplanted host organs and has been well documented following liver transplantation (137). This phenomenon has the ability to facilitate tolerance through deletion of alloreactive T-cells within the thymus and through peripheral effects by interactions between recipient- and donor-derived leukocytes. Whilst there are occasional case reports of complete hematopoietic chimerism occurring post-liver transplant (138), persistence of T-cell chimerism beyond the initial few weeks following liver transplantation is considered unusual (139). Furthermore, even patients with high degrees of chimerism continue to exhibit *in vitro* alloimmune responses up to 1 year post-transplant (140) and may still suffer clinically significant rejection episodes (141, 142). Despite these conflicting data, therapeutic options for inducing chimerism such as combined hematopoietic stem cells and solid organ transplant, thymus transplantation and intra-thymic injection of donor alloantigens, remain an exciting avenue for promoting tolerance (143).

## Future Therapeutic Strategies to Induce Long Term Tolerance

**FIGURE 5:**
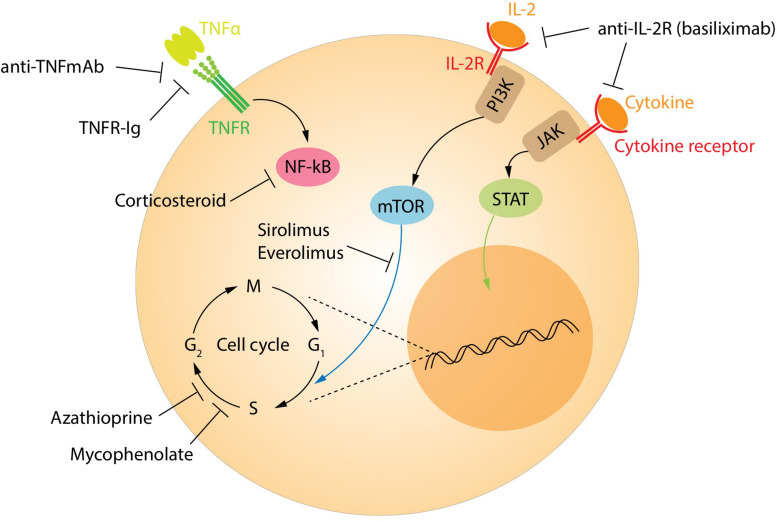
Treatment targets of immunosuppression. The main targets of the immunosuppressive drugs.

The currently recommended immunosuppression regimens have significantly reduced the occurrence of acute rejection and improved outcomes for transplant recipients. However, this comes at the price of increased risk of infections and neoplasia compared with the background population. Currently, patients are treated with a calcineurin inhibitor, either tacrolimus or cyclosporine, along with an antiproliferative drug such as mycophenolate mofetil (Figure 5). These drugs target all T-cell populations and prevent the normal activation and function of both effector and regulatory T-cells. Biological drugs targeting specific pathways continue to be tested in an attempt to reduce the side effects. Some biological agents already in clinical use include the monoclonal antibodies alemtuzumab (anti-CD52) and anti-thymocyte globulin. These drugs broadly target most lymphocyte populations, including regulatory subtypes. Interestingly, Treg and regulatory B-cells are among the first to re-populate the peripheral blood in patients treated with these agents, helping to pushing the balance in favor of the tolerance (144, 145).

Treg-based cell therapy is a promising alternative approach to promote allograft acceptance, potentially minimizing reliance on traditional immunosuppression (146–150). An early approach involved infusing donor antigen-specific Treg and allowed seven out of ten patients to successfully wean from immunosuppression by 18 months post-transplantation (151). Recently data from the ONE study have been published, demonstrating that Treg cell therapy in donor kidney transplant recipients is safe, although missing the efficacy endpoint (152). Other transplant centers have ongoing clinical trials mainly focusing on manufacturing alloantigen-specific Treg (Table 1). This is based on the evidence that alloantigen specific Treg exhibit a better suppressive function toward the alloreactive T effector cells than polyclonal Treg (153, 154). The *in vitro* expansion of the antigen-specific Treg using antigen presenting cells is inefficient due to the small number of cells in the original polyclonal population. A different solution is to engineer human T-cells with genes encoding for the chimeric antigen receptor (CAR). CAR T-cells were approved for clinical usage in 2017 and have since been proved to be effective in cancer treatment and in preventing allograft rejection (155, 156). The *in vivo* expansion of Treg represents another interesting therapeutic strategy. Treg express a higher affinity for IL-2 thus the usage of a very low dose of this molecule can expand the Treg pool *in vivo* up to eight times without significantly increasing the number of T effector cells (157).

**TABLE 1:** Registered clinical trials involving regulatory T-cell therapy in liver transplantation.

Trial	Institution	Phase of the study	Primary outcome	Infused Treg clonality	Number of patients enrolled	Status
Todo/Okomura	Hokkaido, Japan	Phase I/IIA	– Safety – IS weaning – Number of Operationally Tolerant participants	Donor specific	10	Data published (148)
ARTEMIS (NCT02474199)	UCSF, United States	Phase I/II	– Safety – Incidence of AR, CR, re-transplantation, and death – Patients Who Are Able to Reduce CNI Dosing and Discontinue a Second IS Drug with stable LFTs	Donor specific	14	Completed
dELTA (NCT02188719)	UCSF, United States	Phase I	– Safety	Donor specific	15	Terminated
LITTMUS-UCSF (NCT03654040)	UCSF, United States	Phase I/II	– Safety – Number of Operationally Tolerant participants	Donor specific	N.A.	Withdrawn
LITTMUS-MGH (NCT03577431)	MGH, United States	Phase I/II	– Safety – Number of Operationally Tolerant participants	Donor specific	9*	Recruiting
ThRIL (NCT02166177)	King’s college Hospital, United Kingdom	Phase I/II	– Rate of dose limiting toxicities – Graft Loss	Polyclonal	9	Completed
NCT01624077 (First Trial)	Nanjing, China	Phase I	– Patient and graft survival	Polyclonal	1*	Unknown
NCT01624077 (Second Trial)	Nanjing, China	Phase I	– Patient and graft survival	Donor specific	1*	Unknown

From Clinicalgrials.gov, last accessed 19/05/2020. Abbreviations: IS, immunosuppression; AR, acute rejection; CR, chronic rejection; LFT, liver function tests. *Estimated.

In order to suppress T cell proliferation and activation, MSC-therapy based offers an opportunity to promote the tolerance and reduce the immunosuppressive dose in solid organ transplantation. Although the variation in cell product and the heterogeneity of tissue origin makes interpretation of previous clinical studies challenging, MSC therapy is certainly promising. Currently the ongoing mYSTEP1 trial is testing safety and efficacy of donor derived bone marrow MSC pediatric living-donor liver transplantation (158).

Another cell therapy-based strategy has been proposed as immunomodulatory treatment using tolerogenic DC with low expression of MHC I and II and costimulatory B7 molecules and increased expression of PD-L1. These cells are readily derived from fresh or cryopreserved bone marrow derived progenitors (159). The infusion of *ex vivo* donor derived DCreg before transplant was shown to be effective in inducing liver transplant tolerance in murine models, inducing T-cell hyporeactivity thus extending liver allograft survival (160). Mesenchymal stromal cells are also being explored as a potential cell based therapy. These are multipotent cells isolated from tissues such as bone marrow, subcutaneous fat, umbilical cord and tooth pulp, with the ability to suppress immune responses via multiple cell to cell interactions and cytokine release (161). Infusion of these cells has been shown to prevent rejection in liver transplant animal models (162, 163).

Novel therapeutic strategies like *ex vivo* perfusion are already augmenting the pool of transplantable organs (164). Organ reconditioning strategies have already been applied in animal models to reduce the ischemia reperfusion injury by the infusion of MSCs or other anti-inflammatory agents (165) or to reduce steatosis using defatting agents before transplantation (166). Organ machine perfusion opens the door to a different approach to try to induce the tolerance by the infusion of tolerogenic molecules or treating the graft by immunomodulatory cells prior to implantation in the donor.

## Conclusion

Despite major improvements in clinical outcomes following liver transplantation, the majority of patients remain dependent on long term immunosuppressive regimens. This highlights the persistence of alloreactive immunological processes and their tendency to overcome the specific tolerogenic mechanisms of the liver and cause rejection. Further elucidation of the underlying immunology will add to our understanding of this complex phenomenon. Meanwhile, several translational studies, including cell-based therapy approaches, offer the potential of enhancing tolerogenicity whilst avoiding the side effects of current therapeutic strategies.

## Author Contributions

VR and OC collaborated in writing and editing the manuscript and in developing the figures. CM developed the figures and contributed to manuscript writing. GW contributed to manuscript writing. All authors contributed to the article and approved the submitted version.

## Conflict of Interest

The authors declare that the research was conducted in the absence of any commercial or financial relationships that could be construed as a potential conflict of interest.

## Abbreviations

APC: antigen presenting cell
CAR: chimeric antigen receptor
DC: dendritic cell
HLA: human leukocytes antigens
ICAM1: intracellular adhesion molecule 1
IFN- γ: interferon-gamma
KC: Kupffer cell
LSEC: liver sinusoidal endothelial cells
MHC: major histocompatibility system
NK: natural killer
TGF- β: tumor growth factor-beta
Th: T helper cell
Treg: regulatory T cell
VCAM1: vascular adhesion molecule 1.
